# Executive Performance Across Levels of Subjective Socioeconomic Status: Evidence from a Tunisian Population

**DOI:** 10.1192/j.eurpsy.2026.10747

**Published:** 2026-07-31

**Authors:** M. Ben Hmida, D. Le Gall, T. Bellaj, R. Ben Rejeb

**Affiliations:** 1 Psychology department, LPPL lab, Angers university, Angers; 2 Addictology department, EPSM de la Sarthe, Le Mans, France; 3 Laboratoire de Psychologie clinique : Intersubjectivité et Culture, Université de Tunis, Tunis, Tunisia

## Abstract

**Introduction:**

Socioeconomic status (SES) affects cognitive functioning and life outcomes. Most research focuses on objective indicators such as income, education, or occupation. Less is known about subjective socioeconomic status (SSS), i.e., people’s perception of their social position, which may also influence executive functioning, particularly in North African populations.

**Objectives:**

This study examined whether subjective socioeconomic status is associated with executive performance in a Tunisian sample.

**Methods:**

A total of 151 healthy adults from Tunis (Age: *M* = 39.49; *SD* = 13.18; *Range* = 19-74) were recruited. Participants were categorized into three subjective socioeconomic groups (low, low-middle, and high-middle/high) using the MacArthur Subjective Socioeconomic Status Scale. The original design included four groups, but the high-middle and high categories were merged due to the small number of participants in the highest group. Executive functioning was assessed across four domains: inhibition (Stroop, Hayling), cognitive flexibility (Verbal Fluency, Modified Card Sorting Test), working memory (Digit Span, Corsi Block Tapping), and behavioral regulation (BRIEF questionnaire).

**Results:**

ANOVA analyses showed significant differences between groups for both cognitive and behavioral executive processes. Participants in the high-middle/high group consistently outperformed those in the low and low-middle groups across inhibition, flexibility, and working memory tasks, with shorter execution times and fewer errors. They also reported stronger behavioral control and emotional regulation on the BRIEF compared to the low-middle group. These differences remained significant when controlling for intelligence, but not when controlling for objective SES, education, or income.

**Conclusions:**

These findings suggest that subjective socioeconomic status relates to executive functioning, though its effects are intertwined with objective socioeconomic indicators. This study contributes to understanding how socioeconomic perceptions and realities jointly influence cognition in North African populations. Further research may help guide interventions to support cognitive and mental health in individuals facing socioeconomic disadvantage.

**Disclosure of Interest:**

None Declared

